# The complete chloroplast genome sequence of *Smilax microphylla* C. H. Wright

**DOI:** 10.1080/23802359.2021.1947914

**Published:** 2021-07-06

**Authors:** Feng Wu, Daqu Liang, Jingyi Yang

**Affiliations:** a Institute for Forest Resources & Environment of Guizhou, Key Laboratory of Forest Cultivation in Plateau Mountain of Guizhou Province, Guizhou University, Guiyang, China; b College of Forestry, Guizhou University, Guiyang, China

**Keywords:** *Smilax microphylla*, chloroplast genome, phylogenetic analysis

## Abstract

*Smilax microphylla* C. H. Wright is a climbing shrub that can be used for herbal medicine. The complete chloroplast genome sequence of *S. microphylla* was determined in this study. It is 158,246 bp in length with a GC content of 37.12%, and consists of a pair of inverted repeat (IR) regions of 27,175 bp, a large single copy (LSC) region of 85,229 bp, and a small single copy (SSC) region of 18,667 bp. The genome encoded 132 genes, including 86 protein-coding genes, 38 tRNA genes, and 8 rRNA genes. The phylogenetic analysis showed that *S. microphylla* is phylogenetically closely related to *Smilax china* and *Smilax nipponica*.

The genus *Smilax* L. consists of more than 300 shrub species from the Liliaceae family. *Smilax microphylla* C. H. Wright is a climbing shrub that grows at elevations of 500-1600 meters under forest canopies, in brush or on shaded slopes. It is naturally distributed in Guizhou, Sichuan, Hunan, Northeast Yunnan, South Gansu, and West Hubei, China (Fang et al. 2011). *Smilax microphylla* can be used for herbal medicine, providing relaxing effects on tendons and collateral ligaments, acting as an expectorant and relieving coughs (Lin et al. 2012; Liu et al. 2013). However, there is a few research on the genome of *S. microphylla* in current. In this study, we report a complete chloroplast genome of an *S. microphylla* specimen to provide valuable references for the molecular and genomic characterization of *S. microphylla*.

The leaves of *S. microphylla* were collected from Huaxi (Guiyang, Guizhou, China; E:106°65′58″; N:26°44′47″) and deposited in the Institute for Forest Resources & Environment of Guizhou at Guizhou University (http://frerc.gzu.edu.cn, Feng Wu, fwu@gzu.edu.cn, under the voucher number SM-GZU-001). The total genomic DNA was extracted from fresh leaf tissues using DNAsecure Plant Kit (TIANGEN, BJ, China) and sequenced using the Illumina NovaSeq platform; approximately 5 GB of data was generated. The chloroplast was assembled by SPAdes (Bankevich et al. 2012). Moreover, the genome was annotated using CpGAVAS (Liu et al. 2012). The results were compared with published protein-coding chloroplast genomes and rRNA. Blastn and Blastp (https://blast.ncbi.nlm.nih.gov/Blast.cgi) methods were used to verify the accuracy of the results. The annotations of tRNA were carried out with ARWEN (Laslett and Canbäck 2008).

The complete chloroplast genome of *S. microphylla* (GenBank accession MW423607) was 158,246 bp in length, with a GC content of 37.12%. The genome contained a large single-copy (LSC) region of 85,229 bp and a small single copy (SSC) region of 18,667 bp, separated by two inverted repeat (IR) regions of 27,175 bp each. The chloroplast genome was predicted to contain 132 genes, including 86 protein-coding genes, 38 tRNA genes, and 8 rRNA genes. 13 genes contained introns; 11 genes had one intron, and the other 2 genes had two introns.

To explore the phylogenetic status of *S. microphylla*, the chloroplast genomes of 19 species from the Liliaceae family and that of *Arabidopsis thaliana* as an outgroup, were aligned by MAFFT (Katoh and Standley 2013). After that, a phylogenetic tree was constructed by the maximum likelihood method using IQ-TREE (Nguyen et al. 2015) with 1000 bootstraps. The results showed that *S. microphylla* is phylogenetically closely related to *Smilax china* and *Smilax nipponica* (Figure 1).

**Figure 1.**
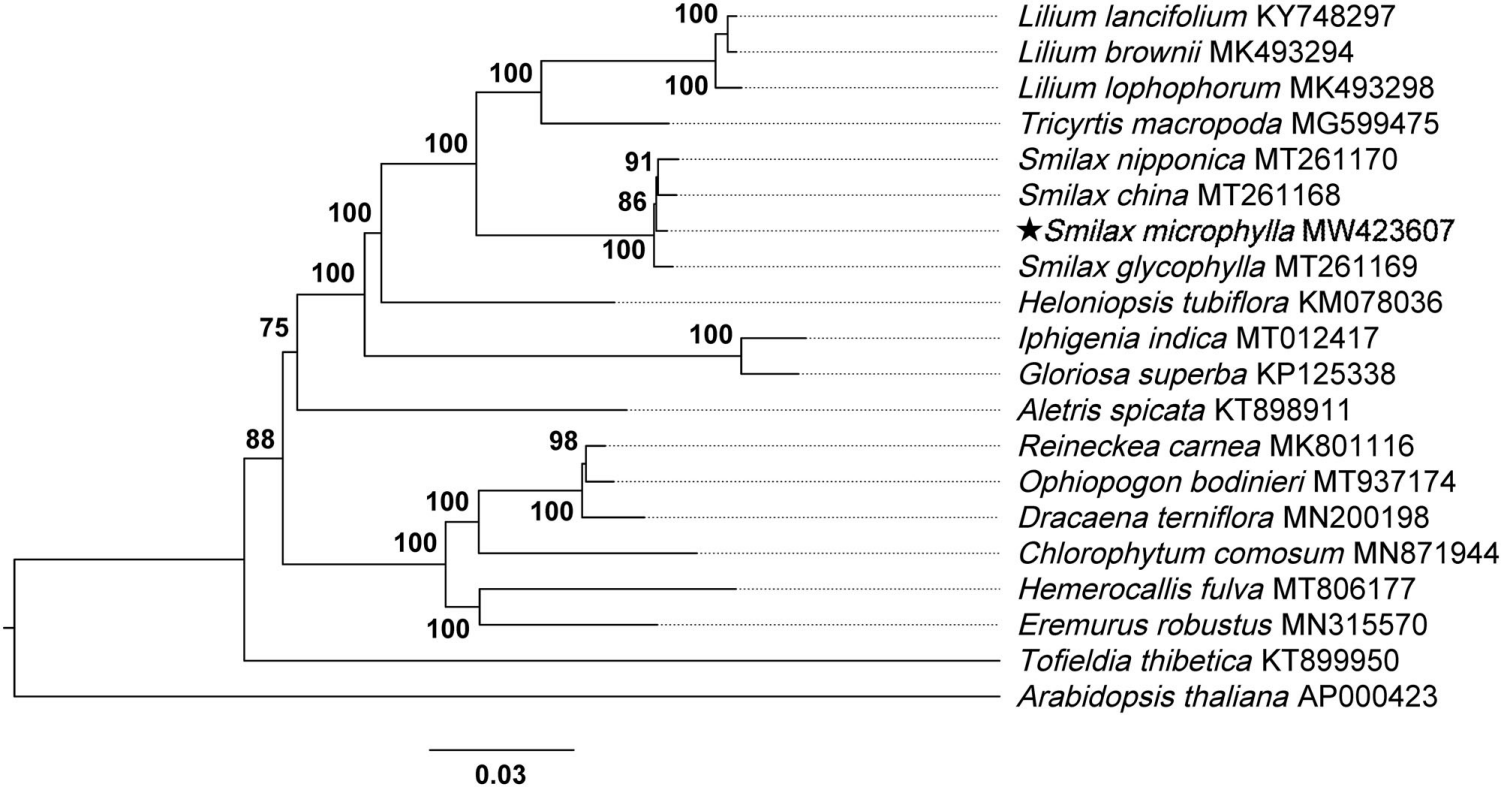
The maximum-likelihood tree of Liliaceae built based on complete chloroplast genomes, with *Arabidopsis thaliana* as an outgroup. The numbers near each node indicate the bootstrap support values.

## Data Availability

The genome sequence data that support the findings of this study are openly available in GenBank of NCBI at (https://www.ncbi.nlm.nih.gov/) under the accession no. MW423607. The associated BioProject, SRA, and Bio-Sample numbers are PRJNA716441, SRS8536284, and SAMN18436054, respectively.
